# The Complexity of Swine Caliciviruses. A Mini Review on Genomic Diversity, Infection Diagnostics, World Prevalence and Pathogenicity

**DOI:** 10.3390/pathogens11040413

**Published:** 2022-03-29

**Authors:** Irit Davidson, Efthymia Stamelou, Ioannis A. Giantsis, Konstantinos V. Papageorgiou, Evanthia Petridou, Spyridon K. Kritas

**Affiliations:** 1 Division of Avian Diseases, Kimron Veterinary Institute, Bet Dagan 50250, Israel; davidsonirit@gmail.com; 2 Department of Microbiology and Infectious Diseases, Faculty of Veterinary Medicine, School of Health Sciences, Aristotle University of Thessaloniki, 54124 Thessaloniki, Greece; efistamel@hotmail.gr (E.S.); pgkostas@yahoo.gr (K.V.P.); epetri@vet.auth.gr (E.P.); skritas@vet.auth.gr (S.K.K.); 3 Department of Animal Science, Faculty of Agricultural Sciences, University of Western Macedonia, 53100 Florina, Greece

**Keywords:** molecular identification, recombinant, pigs, diarrhea, asymptomatic

## Abstract

Caliciviruses are single stranded RNA viruses, non-enveloped structurally, that are implicated in the non-bacterial gastroenteritis in various mammal species. Particularly in swine, viral gastroenteritis represents a major problem worldwide, responsible for significant economic losses for the pig industry. Among the wide range of viruses that are the proven or suspected etiological agents of gastroenteritis, the pathogenicity of the members of Caliciviridae family is among the less well understood. In this context, the present review presents and discusses the current knowledge of two genera belonging to this family, namely the *Norovirus* and the *Sapovirus*, in relation to swine. Aspects such as pathogenicity, clinical evidence, symptoms, epidemiology and worldwide prevalence, genomic diversity, identification tools as well as interchanging hosts are not only reviewed but also critically evaluated. Generally, although often asymptomatic in pigs, the prevalence of those microbes in pig farms exhibits a worldwide substantial increasing trend. It should be mentioned, however, that the factors influencing the symptomatology of these viruses are still far from well established. Interestingly, both these viruses are also characterized by high genetic diversity. These high levels of molecular diversity in Caliciviridae family are more likely a result of recombination rather than evolutionary or selective adaptation via mutational steps. Thus, molecular markers for their detection are mostly based on conserved regions such as the *RdRp* region. Finally, it should be emphasized that *Norovirus* and the *Sapovirus* may also infect other domestic, farm and wild animals, including humans, and therefore their surveillance and clarification role in diseases such as diarrhea is a matter of public health importance as well.

## 1. Who Is Who among Swine Enteric Infections?

Viral gastroenteritis is a serious disease in pigs, with high morbidity observed worldwide, responsible for significant financial losses. Keeping this in mind, surveillance and characterization of pig enteric viruses is essential to evaluate possible animal health risks as well as for the epidemiological analysis and determination of economic impacts on pig farming and the meat industry.

At least 11 enteric viruses belonging to 6 distinct families (Adenoviridae, Astroviridae, Caliciviridae, Coronaviridae, Parvoviridae, and Reoviridae) cause non-bacterial diarrhea in swine, mainly during the nursing and immediate post-weaning period. The swine enteric viruses include transmissible gastroenteritis virus (TGEV), porcine epidemic diarrhea virus (PEDV), rotavirus, astrovirus, sapovirus (SaPV), norovirus (NoV), kobuvirus and other agents [1,2]. Most viruses infect the small intestinal enterocytes, causing various degrees of villous atrophy and subsequently a malabsorptive, maldigestive diarrhea. In addition, concurrent infections with multiple enteric viruses can trigger synergistic or additive effects, leading to more extensive villous atrophy throughout the intestine and more severe and prolonged diarrhea [1]. Numerous studies have been published towards the swine enteric virus complex investigation, and given their high number and complexity, most of them focus on only several swine enteric viruses each time, not covering all the enteric viruses. Accordingly, the present review is focused on the Caliciviridae family, and particularly on two among its eleven genera, namely the genus *Norovirus* and the genus *Sapovirus*. The other nine genera are the *Lagovirus*, *Vesivirus*, *Nebovirus, Recovirus, Valovirus, Bavovirus, Nacovirus, Minovirus and Salovirus* [2]. Viruses within *Lagovirus*, *Norovirus*, *Nebovirus*, *Recovirus*, *Sapovirus*, *Valovirus* and *Vesivirus* genera infect a wide range of mammals; members of Bavovirus and Nacovirus infect birds; and members of *Minovirus* and *Salovirus* infect fish [3,4,5,6,7]. Caliciviruses have also been detected in the greater green snake and frogs [8]. 

**Figure 1:**
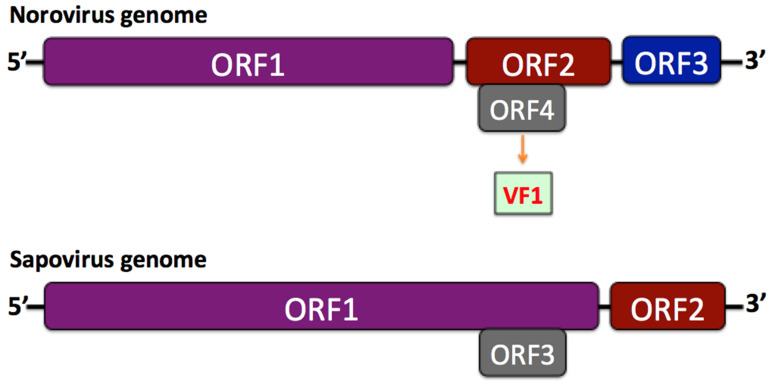
Comparative structure of *Norovirus* and *Sapovirus* genomes.

The caliciviruses are non-enveloped, single-stranded, positive-sense RNA viruses, approximately 7.3–8.5 kb in size. Based on their genome structure, the Caliciviridae family can be further differentiated into two groups [9]. In the first, including the *Norovirus*, the open reading frame 1 (ORF1) is separated from ORF2 and ORF3 near the 3′ end, while an ORF4 (comprised within ORF2) encodes the virulence factor, VF1 (Figure 1). In the second group, containing the *Sapovirus*, there is a large ORF1 and a standard ORF2 (equivalent to ORF3 of the *Norovirus*), whereas an ORF3, equivalent for ORF4 was suggested (Figure 1).

## 2. The Elusive Clinical Evidence of Calicivirus Infection in Commercial Pigs

Porcine SaPVs infect pigs of all ages, causing diarrhea particularly in young ones, whereas infection with porcine NoVs has been referred only in adult pigs, mostly not associated with clinical signs [10]. Successively, NoVs were found to be circulating in healthy adult pigs [11]. Salamunova et al. [12] detected an equal rate of SaPVs prevalence in Slovakian clinically healthy and in diarrheic pigs (8.4% and 10.0%, respectively). In recent years, increased awareness of asymptomatic NoVs infections and the potential of infected pigs to be a reservoir for the emergence of new viruses has been raised.

In general, the swine infection with enteric caliciviruses is often asymptomatic, and these viruses could express their pathogenicity in only a small percentage of cases due to co-infection with other gastrointestinal viruses or owing to immunodeficiency of animals. In swine, co-infections with the ubiquitous immunosuppressive circovirus Porcine Circovirus-2 (PCV-2) [13] might augment the pathogenicity of other pathogens, including enteric caliciviruses, as it performs in the Postweaning Multisystemic Wasting Syndrome and Porcine Dermatitis and Nephropathy Syndrome, i.e., the porcine circovirus disease (PCVD). Concerning many viruses, it is believed that diarrhea development in pigs is a result of interaction of the entire virome or microbiome with the microenvironment within the intestinal tract, which synergistically, may influence the course of viral infection [14].

Even though the NoVs have wide existence worldwide in the pig population, their detection rate is relatively low, between 0–16.6% without outbreaks, but mostly with asymptomatic infections [15]. These inferences intensify the vulnerability of human exposure and infection with the swine zoonotic enteric viruses, since exposure may occur accompanied with no notable sign, and even without any awareness. Following the realization that swine caliciviruses might be zoonotic pathogens and may be present in asymptomatic commercial pigs, these viruses retain an important position within the ONE HEALTH area. ONE HEALTH represents a holistic approach concerning food production, anthropozoonoses control and antibiotic resistance, towards common policies, legislation and research design by all related stakeholders.

Sapovirus infections are also common in swine worldwide. Lauritsen et al. [16] detected SaPVs in pigs of all age groups, but most frequently in post-weaning pigs. The SaPVs were classified to belong to genogroups GIII (strain A, B, and C) and GVII. At 5½ weeks of age the SaPVs were detected in 82% of the pigs in Group A and in 68% of the pigs in Group B, but they could not be recovered in the second sampling that was performed when the pigs were 15–18 weeks old. However, Lauritsen et al. [16] found in the second sampling that 13 of the 51 pigs excreted another sapovirus strain, genogroup GIII (strain D). Since more pigs were SaPV-positive at 5½ weeks of age as compared to 15–18 weeks, it seems that pigs are transient shedders of SaPVs, confirming that SaPVs infections are highly prevalent in post-weaning pigs. Dufkova et al. [17] demonstrated that the SaPVs of genotype III prevail in post-weaning pigs from Czech farms at a 28.3%, and their prevalence was significantly higher than in finisher pigs (9.8%) and in nursing piglets (3.0%), while the sows were negative for SaPVs, probably because of the virus neutralization provided by the maternal antibodies.

## 3. The Worldwide Prevalence of Swine Calicivirus in Commercial Pigs

Since the first discovery of NoVs in 1972 [18] and its characterization as the cause of human diarrhea (designated as the Norwalk prototype), it took about 25 years until the first description of NoV infection of pigs in Japan, and later in Europe and the US [19,20,21]. The SaPVs were also first detected in humans during an infant gastroenteritis outbreak in 1977 in Sapporo, Japan [22]. Three years later, in 1980, Saif et al. [23] described the first porcine SaPV (the Cowden strain) in a co-infection with rotavirus and astrovirus particles by electron microscopy. 

The swine NoVs and SaPVs have been detected and described in many parts of the world (Table 1). Keum et al. [24] described the circulation of porcine NoVs (GII) and SaPVs (GGIII) in Korea by surveying 537 fecal samples. The rates of both viruses were 1.9% and 11.2%, respectively, and no co-infection by both viruses was detected. Keum et al. [24] study is the second report from Asia regarding the detection of swine caliciviruses, after Japan, although the report from Japan appeared one year later [25]. In Japan the porcine and human SaPVs were genetically similar, belonging to GIII and GV, suggesting potential zoonotic transmission. In their study, Nakamura et al. [25] described co-infection of 20 pigs with NoVs and with SaPVs, rendering opportunities for genetic recombination between various viruses. In total, 240 pigs were tested for the presence of NoV and SaPV. Notably, all infected pigs were asymptomatic.

The porcine caliciviruses’ wide spread was demonstrated by Cunha et al. [26] who described for the first time the NoV GII.18 clade in Latin America in one stool sample, after examining 96 fecal samples from pigs of different ages from five farms from Rio de Janeiro State, Brazil. The infected animal was a healthy finisher pig. In Central and Southern Taiwan, 533 pig fecal samples from six farms were tested for the presence of Caliciviruses using RT-PCR. NoVs and SaPVs were detected in pig fecal samples at a positive rate of 7.1% and 0.6%, respectively [27]. In the same study, the differences in porcine Norovirus prevalence in relation to season were evaluated for the first time, indicating a higher positivity rate in winter, i.e., 41.7%, as compared to the 26.4% observed in the summer. Shen et al. [28] reported the first recombinant new genotype NoV in a pig herd in China, after examining 12 fecal samples from piglets with diarrhea. Two of the twelve examined samples were positive for PoNoVs, one of which was positive for PoNoV alone, and the other was coinfected with porcine Circovirus and PoNoV. The new NoV genotype that was detected belonged to the sample in which PoNoV was detected alone. In China, Jun et al. [29] described piglets’ infection with SaPVs of genotypes GIII and GVI. The detection rate of SaPV was 3.4% (5/146). Scheuer et al. [30] surveyed the NoVs in the North Carolina swine population using 413 pooled fecal samples from apparently healthy finisher pigs in 2009 and found about 18.9% positivity using RT-PCR coupled with hybridization assay. In the same time period, a similar NoV prevalence of 20% was recorded in finisher pigs of three U.S. states, including North Carolina [31]. In this study of Wang et al. [31], 621 fecal samples were collected from swine of various ages from 7 swine farms and 1 slaughterhouse in three states in the United States. Fecal samples were tested by reverse transcription-PCR and microwell hybridization assays with porcine NoV- and SaPV-specific primers and probes, respectively. The same study also reported the detection of porcine GIII SaPVs in 62% of pigs, with the highest prevalence in postweaning pigs and lowest in nursing pigs. In Canada, a 25% (30/120 swine fecal samples tested positive) prevalence of NoV was found [32]; however, a lower NoV prevalence was detected in Europe, namely ranging between 2–4.6% positivity [33,34]. More specifically, in the study of Mijovski et al., 2010, where 406 swine fecal samples from 8 pig farms were tested for the presence of Caliciviruses, 5/406 (1.2%) of the samples tested positive to NoV by RT-PCR and sequencing, while 29/406 (7.1%) of the samples were positive to SaPV [33]. In the study of Mauroy et al. [34] in Belgium, 43 swine fecal samples from a veterinary diagnostic laboratory were examined and PoSaPVs were detected in 5/43 stool samples of both diarrheic and asymptomatic piglets, while Porcine NoVs were only detected in 2 pigs without clinical signs. PoNoV strains were detected in younger pigs (16–20 weeks) [34]. In New Zealand, GII NoV was detected in 2/23 (9%) of porcine specimens examined using a multiplex real-time RT-PCR [35]. NoV prevalence was 8% in Brazil [26,36] and 1–15% in Asia [25,27,37]. NoVs of genotype II were also detected in pigs at slaughter in Germany [38], Brazil [39] and Ethiopia [40]. Serological evidences indicated that NoVs are circulating also in rural Nicaragua [41].

Dufkova et al. [24] demonstrated that asymptomatic pigs carry GIII SaPVs at a rate of 10.25% (20/196) in Czech pig farms, in mixed infections with astroviruses (34.4%) and kobuviruses (87.3%), which are the most prevalent swine enterovirus infection agents. The SaPVs positivity rate in pigs in Hungary was 17.6%, (3/17 samples), while in China, Korea and the USA, the positivity rates were 8.1% (8/99), 29.1% (69/237) and 9% (35/377), respectively [21,42,43,44]. More recently, Di Bartolo et al. [45] described the first detection of porcine NoV in Northern Italy. In this study, 201 fecal specimens from asymptomatic and 89 specimens from pigs with diarrhea were examined for the presence of porcine Caliciviruses and PoSaPV was detected in 6.9% of the asymptomatic pigs and in 18/89 (20%) of the symptomatic pigs, while PoNoV was detected in 1 asymptomatic pig [45]. The Italian NoVs were genotyped as GII.11 and prevailed in asymptomatic pigs at a rate of 0.5%. Interestingly, in another survey performed in Italy a year later, no positive samples for NoVs were detected in a total of 242 swine fecal samples examined [46], suggesting that the rate of virus circulation is variable. 

Salamunova et al. [12] documented the molecular detection and diversity of enteric viral agents in suckling, weaned and fattening pigs on farms in Slovakia. PAstV was found to be the dominant virus species with high prevalence (80–99%) in the investigated farms, but its presence did not depend on the health status of pigs. On the other side, porcine SaPVs were found in a small percentage (around 9% (37/411)) in both healthy and diarrheic animals, with higher occurrence in suckling piglets. The equal presence of both viruses in healthy and diarrheic pigs does not clearly clarify their role in gastrointestinal diseases and therefore detection has diagnostic value only in conjunction to clinical signs. Valko et al. [47] conducted an extensive survey in an attempt to identify the diarrhea-related porcine viruses, including adeno-, astro-, boca-, calici-, circo-, corona-, kobu-, rota- and Torque teno viruses, by examining a total of 384 fecal samples from 17 farrow-to-finish pig farms. Regarding the animals’ health status, 239 of these samples derived from diarrheic pigs and 145 derived from asymptomatic animals. Caliciviruses were detected in six farms, with a percentage of 5.6% [47]. The results of this study suggested that the complexity of this disease may vary with age, which makes the prevention of diarrhea a challenge, especially in weaned pigs. Additionally, in Cavicchio et al.’s study [48] in North East Italy, 225 swine fecal samples from 74 swine herds in Veneto region were examined for the presence of NoVs, which were identified in 11.4% of the analyzed samples [48]. NoV was mainly detected in fattening pigs and a co-circulation of diverse swine NoVs subgroups was demonstrated, thus raising concern on the emergence of potentially zoonotic viruses by recombination events. Caliciviruses have also been detected in swine in Greece, when 1400 porcine fecal samples from asymptomatic pigs of 5 different age groups from 28 pig farms around Greece were examined with two molecular assays, i.e., conventional and SYBR-Green Real-time RT-PCR [49]. In this study, the p289-p290 primer pair was used for the detection of Caliciviruses in both conventional and Real-time RT-PCR, targeting the RdRp conserved region of Caliciviruses, which creates an amplicon of 331 bp for Sapovirus and an amplicon of 318 bp for Norovirus. Caliciviruses were detected in 12.9% and 20.4% of the examined pools of samples with the method of conventional and SYBR-Green RT-PCR, respectively. These differences were most likely attributed to the nature of the molecular methods, with Real-Time PCR being generally more sensitive in microbes and parasites detection [50]. The age group distribution of Caliciviruses in the aforementioned epidemiological study was 10.7% at suckling pigs, 8.9% at nursery pigs, 12.5% at grower pigs, 30.4% at finishing pigs and 1.8% at sows, based on the results of conventional RT-PCR. Based on the results of the SYBR-Green real time RT-PCR, Caliciviruses were also prevalent in finishing pigs (64.3%). Moreover, two SaPV sequences were acquired after sequencing of the positive samples and phylogenetic analysis revealed the close genetic similarity of these sequences with porcine SaPVs sequences from China and Brazil [49].

In general, in the majority of the studies where both NoV and SaPV were investigated, co-infection occurred in very low prevalence (Table 1). This is in line with the general asymptomatic nature of those viruses that may mostly be diarrheic in the context of multiple infections, along with other microbes. Considering the prevalence data recorded for both viruses, demonstrating a recent (in approximately the last two decades) global wide distribution in hosting pigs, we can conclude an intensively increasing trend in swine, which is enhanced by their asymptomatic nature that assists spread and fast transmission. It should be also noted that this increasing trend reflects and is supported by the improved detection methodologies available.

**Table 1:** Worldwide infection rates of NoVs and SapVs in swine.

Region	NoVs	SapVs	Co-Infection of Enteric Viruses (NoVs and SaPVs)	Sample Size	Year of the Study	Season	References
USA	20%	62%	5.4%	621	2002–2005	All year	[31]
Canada	25%	not examined	-	120	2005	Autumn	[32]
South Korea	not examined	29.1%	-	237	2004–2005	All year	[42]
Hungary	5.9%	11.8%	-	17	2005	Spring	[43]
New Zealand	9%	not examined	-	23	2006–2007	All year	[35]
Korea	1.9%	11.2%	-	537	2007–2009	All year	[24]
Brazil	1%	-	-	96	2007	Summer	[26]
Japan	16.7%	33.4%	0.08%	240	2008	All year	[25]
China	not examined	8.1%,	-	19	2008	Winter	[44]
Taiwan	7.1%	0.6%	0.2%	533	2008	-	[27]
North Carolina, USA	18.9%	-	-	12	2009	Summer	[30]
Czech Republic	not examined	10.25%	-	196	2010–2011	All year	[17]
Italy	0.5%	11%	-	290	2006–2007 and 2012	All year	[45]
Italy	-	not examined	-	242	2012–2014	All year	[46]
Slovakia	not examined	9%	-	411	2013–2016	All year	[12]
East Italy	11.4%	not examined	-	225	2018–2019	All year	[48]
Greece	-	64%	-	280	2019	All year	[49]

## 4. The Extreme Swine Calicivirus Molecular Diversity

NoVs and SaPVs are genetically highly diverse viruses. The Norovirus genus were previously classified into at least 10 genogroups that were further classified into more than 40 genotypes [15]. The SaPVs are even more diverse, containing 14 genogroups based on the VP1 gene sequence [16,51]. Recently, the taxonomy of SaPVs has been revised and classified into 19 genogroups (G) and at least 52 genotypes based on complete VP1 sequences [52,53]. This classification was accomplished using a pairwise distance cut-off value of ≤0.488 in order to distinguish different genogroups and ≤0.169 to distinguish different genotypes [52,53]. This is particularly notable considering their relatively small genome size that does not exceed 8.5 kb for SaPV and ∼7.5 kb for NoV, which falls within the average thresholds for non-enveloped viruses. It is generally accepted that between genomic size and mutational rate, an inverse relationship occurs, enhancing the genetic diversity in larger-sized, usually enveloped viruses [54]. Similarly, virion architecture is directly correlated with genomic diversity, a correlation characterized by an allometric scale. Under this prism, as described in detail in the following paragraphs, selection and subsequent mutational steps probably do not constitute the main drivers of genomic diversity in caliciviruses; rather, recombination does.

At the time of writing this review article, about 600 sequences of swine caliciviruses are available in the GenBank database (223 of NoVs, 354 of SaPVs and 19 of swine caliviruses), demonstrating their immense genetic variability. As previously mentioned, the extensive genetic diversity among noroviruses is created not only by the occurrence of genetic mutations, with a typical high rate as for highly mutating RNA viruses, ranging between 10^−2^ to 10^−5^ mut/nt/rep [55], but to a greater extent by intra-genomic recombination. Recombination is a driving force of viral evolution and it has been described for many single-stranded RNA viruses, including NoVs such as the new genotype NoV described in China [28]. Recombination in influenza viruses increases the biological fitness and pathogenicity [56]. Moreover, natural processes of molecular recombination among additional virus families, such as among DNA viruses and retroviruses, or within DNA viruses or within retroviruses, the genetic diversity increases the viral diversity [57]. Molecular recombination occurs between the NoVs ORF1 and ORF2 [58]. Processes of recombination between ORF1 and ORF2 of NoVs occur frequently, causing an increased genetic variability. The junction point of ORF1 and ORF2 is referred as “hot spot” [59]. The subgenomic RNA, i.e., a positive-sense molecule located at the 5′ of the capsid gene, is the most likely factor responsible for recombination [60]. This molecule is co-terminus with the virion genome, and when the latter contains genomic and subgenomic RNAs, in case of co-infection, this phenomenon may cause recombination. In general, mechanisms that may favor recombination in caliciviruses implicate errors in RNA polymerism that may be most likely attributed in RNA polymerase.

Porcine and wild boar SaPVs are classified into 8 genogroups and 21 genotypes (GIII, GV.3, GV.5, GVI.1-3, GVII.1-6, GVIII.1-2, GIX.1-2, GX.1-2, GXI.1-3) [53,61]. By December 2019, 26 complete porcine SaPV genomes (11 GIII, 4 GV, 3 GVI, 3 VII, 1 GVIII, 2 GX, and 2 GXI) were available in DDBJ/EMBL/GenBank databases, while the complete genome of a GIX SaPV has not been reported [53]. Porcine SaPVs mainly belong in GIII [62]. For example, the Czech SaPVs were characterized molecularly and subsequently classified to GIII, but they differed molecularly within this genogroup, leading to great amino acid sequence diversities. The amino acid identity ranged between 57.9 to 99.1% [17]. Similarly, the Slovakian SaPVs were molecularly classified to GIII [12], which is the most prevalent genogroup worldwide. Porcine GV SaPVs are genetically closely related to human GV SaPVs. However, to date, there has not been reported any zoonotic transmission of the same genotype of SaPV between pigs and humans [53]. Porcine SaPVs GVI, GVII, GX, and GXI share more common genomic features than other genogroups of SaPVs. GVI, GVII, GIX, GX, and GXI SaPV strains form a unique clade that consists of only porcine and wild boar SaPVs and they are distantly related to other porcine SaPVs (GIII, GV and GVIII) in both trees, based on phylogenetic analyses performed by using nucleotide sequences of complete genomes and VP1 sequences [53].

In the first reported recombinant swine NoV isolate, discovered in China [63], namely the QW170 and QW218 recombinant strains, the breakpoint is located in the RdRp capsid gene junction region. The NoV GII.4 genotype has been predominantly identified by Siebenga et al. [64], who proposed an evolutionary model through accumulation of mutations and recombination events. Co-infection with several NoVs belonging to different genotypes are common [65,66]. The RdRp-capsid junction region of calicivirus contains a highly conserved motif of 20 nucleotides. This conserved nucleotide motif is almost identical within each genogroup of NoVs and SaPVs, enabling homologous recombination during co-infection of a cell with different NoVs and SaPVs. Recombinant caliciviruses having a recombination site at the RdRp capsid junction region were identified in humans, calves and pigs [21,67,68,69]. Similarly, recombination at the RdRp-capsid junction region also occurs among recombinant strains of SaPVs in human [70,71].

## 5. The Current Techniques to Demonstrate Calicivirus Infections

The classical method of virus identification, virus isolation in tissue cultures—which is laborious, expensive and long, with a turnaround time of 28–40 days—does not constitute a diagnostic option for NoVs and SaPVs. Most viruses are difficult to grow in vitro. The porcine SaPV (Cowden strain) can be propagated in LLC-PK cells in the presence of intestinal content or bile acids [72], but attempts to cultivate human SaPVs in cell culture had been unsuccessful [73] until recently, when human SaPV was replicated in human cell lines supplemented with bile acids [74]. The human cell lines originated from testis and duodenum and more efficient virus replication was noticed in the duodenum cell line. Because there is no cell culture system or small animal model for NoVs and SaPVs, except the porcine SaPV, Cowden and the murine NoV MNV-1 strains [75,76], the antigenic classification of these viruses by two-way cross-neutralization tests is not possible. At this point, it should be noted that human Norovirus has been cultured in human B cells [77] with the aid of commensal bacteria that served as a cofactor for the infection. Additionally, cultivation of multiple HuNoV strains in enterocytes in stem cell-derived, non-transformed human intestinal enteroid monolayer cultures has been reported [78]. Finally, replication of HuNoV GI and GII in high titers in zebrafish (*Danio rerio*) larvae has been recently reported [79]. The virus replication was noted to peak at day 2 post infection and the virus was detectable for at least 6 days [79].

The other option for calicivirus diagnosis is virus visualization using electron microscopy; however, that assay is expensive, of low sensitivity and requires a specific expertise grade as well as substantial technical skills of the operator. The antibody demonstration after infection by ELISA is an indirect assay that depends on the animal immune response to infection, and is also laborious, time consuming, of limited availability and is prone to false negative reactions. IFA and antigen-ELISA with virus-specific hyperimmune antisera has been developed to detect GIII Cowden capsid proteins in experimentally infected pigs [75]. Antibodies against porcine SaPVs could be detected in pig serum samples infected with SaPV, using GIII SaPV-specific VP1-ELISA [29,80,81] or recombinant porcine SaPV viral-like particle ELISA [82,83]. However, the above assays lack sensitivity when compared with the detection methods that target viral nucleic acids [52].

Hence, the molecular techniques are the most straightforward and informative tools to demonstrate calicivirus infections. Various methodologies based on conventional and real-time PCR assays have been developed towards this direction, some of which constitute the most common diagnostic tests. The most widely accepted molecular markers for genetic classification of these viruses are those targeting capsid genomic sequences, based on which, phylogeny has been reconstructed [67,84,85]. More specifically, Katayama et al. [67] investigated which genomic region was the most suitable to classify the NoVs. They proposed that the full genome, the complete ORF1 and ORF2 and the capsid N-terminal/S domain could segregate viruses into genus, genogroup and genotypes. However, the RNA-dependent RNA polymerase (RdRp) genes, located within ORF1, represent the most conserved genes in NoVs and SaPVs, the sequence of which could not however assign viruses to genogroups. 

The RT-PCR is the principal assay for detection of porcine NoVs and SaPVs [11,21,31]. The genetic diversity of porcine NoVs and SaPVs is a factor that causes difficulties in selecting PCR primers for the detection of the circulating strains. For that reason, in most studies primers were designed targeting the most conserved RdRp region of the genome, or based on “universal” primers [11,31,62,86,87]. Additionally, the RdRp-capsid junction region [80,88] and the partial capsid region [89,90] have also been utilized for porcine SaPV detection. In this context, a broad range calicivirus primer pair p289/p290 (5′-GATTACTCCAAGTGGGACTCCAC-3′/5′-TGACAATGTAATCATCACCATA-3′), which may be also characterized as the “gold standard” detection method, derived from the RdRp gene and targeting the conserved motifs “DYSKWDST” and “YGDD”, was utilized in a real-time amplification embedding the SYBR Green fluorescence. This assay was reported by Mauroy et al. [34,62] as a first-line fast and sensitive screening assay for porcine caliciviruses. More sensitive and reliable techniques based on real-time amplification have been also developed using TaqMan probes [91] where the fluorescence is on the oligonucleotide instead of being mixed with other PCR components, providing higher reliability by reducing the non-target amplified products that may mislead the results. However, the cost of the probes is only affordable and cost effective for very large numbers of samples or continuing monitoring. It should be emphasized that the genetic allocation of the calicivirus positive samples has to be further confirmed by sequencing followed by BLAST comparisons and phylogenetic analysis, or alternatively melting curve temperature analysis. Sanger-sequencing of RT-PCR products amplified using calicivirus universal primers that target the most conserved regions, such as RdRp, has the advantage of identifying new calicivirus sequences [21,30,37,92,93,94,95]. Some other techniques that have been used more often in recent years for the detection of porcine SaPV sequences in the fecal samples, due to the advances in the metagenomic field, are deep sequencing and next-generation sequencing (NGS) [96,97,98,99,100]. These techniques contribute to the classification of SaPVs, based on entire genomes, and the discovery of new SaPV genotypes [98,100] but fail to discover the complete novel viral sequences because they need a template to assemble the short sequence fragments. Although sequencing constitutes the most reliable and unambiguous validation of positive examined samples, in surveillance or monitoring programs, where fast results are needed, one-step endpoint molecular techniques are generally preferable. Eventually, both SYBR green Real time and TaqMan offer the opportunity to quantify the detected virus, with the precondition of a standard curve available, i.e., a sample of known quantity.

The use of fluidic bead-based technology and tagged primers (xMAP & xTAG by Luminex^®^) [101] is one of the latest developments for detecting simultaneously multiple pathogens, up to 50 using MagPix instrument. Nevertheless, in this case, often genogroups are difficult to be identified, a scope that could be more succesively achieved after sequencing and phylogenetic analysis that considers multiple nucleotide polymorphisms and therefore not affected by single mutations as may sometimes be the case when using tagged primers. High throughput setups using 96-well microplates are suitable for surveillance. The fast turnaround time (5–6 h) and the reduced cost/test/sample is a considerable advantage. A multiplex assay for the detection of six swine enteric viruses was developed using the Luminex fluidic bead-based technology. The assay detected as few as 10 copies of viral nucleic acids of each targeted virus in fecal samples.

## 6. The Interchanging Hosts of Swine Calicivirus

Caliciviruses contained in the two genera *Norovirus* and *Sapovirus* infect pigs and a broad range of hosts, including humans [10]. The susceptible animals include livestock, pets and also numerous wild animals, such as marine mammals and bats. Nevertheless, NoVs genogroup II has been evinced to infect both humans, pigs and dogs, whereas there is no evidence implying that the same genogroups of sapoviruses infect humans and pigs.

More specifically, by genotyping it was revealed that the NoVs are not host restricted and most probably jump across species barriers [15]. NoVs genogroups GI, GII and GIV are infecting humans, but are prevalent also in pigs, dogs and cats. However, cluster GII is prevailed mostly in pigs, whereas cluster IV in dogs and cats. NoVs belonging to other genogroups infect a broad range of hosts, including cows, sheep, marine mammals and rodents. The dogs, especially the dogs that are kept in close proximity with pig farms, aid the danger of spread of the zoonotic NoVs from asymptomatic pigs to humans, as the dogs can be infected by human NoVs. Although dog infection with porcine NoVs has not been reported, there is a possibility that they serve as an intermediate host for reverse zoonosis between humans and pigs, since canine NoVs have been isolated to a great extent from symptomatic versus non-symptomatic animals [102].

The detection of novel strains of NoVs and the detection of human-like NoVs strains in stool samples of symptomatic and asymptomatic farm animals indicated that these animals were the reservoir of the NoVs emerging strains and are of zoonotic potential [19,20,103,104]. These inferences highlight the high importance of early detecting NoV in swine, which although are sometimes likely to not cause any disease, may serve as bridge for human infection. In humans, NoV diarrhea could be therefore characterized as an occupational disease. Domestic animals’ NoVs are genetically similar to the human NoVs, especially those classified in the genotypes GII as GII.11 (prototype SW918). NoVs classified as belonging to GII.18 and GII.19 have been found in stools of pigs in Europe and North and South America and Asia. NoVs were also detected in humans in the African Bobo Dioulasso, Burkina Faso [105]. 

On the other hand, although SaPVs have been detected in various mammal hosts, these animals are most likely asymptomatic to this virus. For instance, about 21.2% from stool samples of symptomatic patients were SaPVs positive, as compared to a similar rate of 24.8% from the samples of asymptomatic patients. Particularly SaPVs are occasionally present in pigs, mink, dogs, sea lions and bats, and particularly SaPVs belonging to genogroups I, II, IV and V (GGI, GGII, GGIV and GGV) are present in humans. With the exception of some particular human age groups, i.e., under 5 years old and over 60 years old, no pathogenicity is usually observed in any of those hosts. The significance of detecting this virus is still high, considering that co-infection with other viruses may result in increased vulnerability for enteric diseases. 

In conclusion, as genetically similar ΝoVs were identified in animals and in humans, these viruses were recognized as possessing anthropozoonotic potential. Therefore, continuous monitoring and virus characterization are needed to detect the infection sources in order to control the infection and to avoid its circulation to humans, not only with respect to livestock, but from a public health point of view as well. Given the increasing epidemiological trend of both swine NoV and SaPV in pig farms worldwide, the high levels of genetic diversity and recombination, and the fast and easy transmission favored by the asymptomatic nature, refined molecular diagnostic tools are essential for the continuing surveillance of these viruses in farm pigs and wild boar.

## Data Availability

Not applicable.
